# Annotations

**Published:** 1903-08-15

**Authors:** 


### ANNOTATIONS.

### Prehistoric Sculptures.

It will be remembered that, something like two years ago, Mr. Auberon Herbert addressed a letter to the *Times* paper, in which he described what he called the "sculptured stones" existing, in great abundance, in some of the gravel pits of the New Forest; and he followed up this communication, last month, by an exhibition of a very large number of these stones, and by a lecture upon them at the Langham Hotel. A good many geologists, we believe, are disposed to regard the resemblances to natural objects, or to human faces, which Mr. Herbert's stones display, as merely fortuitous, and to dismiss the subject from further consideration. No doubt such fortuitous resemblances may occur. We constantly trace them in the fire, or in the formation of clouds, and all travellers are familiar with them on a large scale in natural objects in various places, as in the "Lion" and the "Eagle" rocks in the Straits of Bonifacio; although, in the majority of such examples, it may be doubted whether we have not suggestion rather than actual likeness. But it is also true that men in a low stage of civilisation have not only recognised these resemblances, but have sought to heighten them by artificial means. In many of the islands of the Malay archipelago, for example, the natives are very skilful in manufacturing toys out of pieces of bamboo root; but they always select the piece with a view to the nature of the object which it is ultimately to represent. Mr. Herbert is of opinion that something analogous has been done by the prehistoric men whose works he believes himself to have discovered. He thinks that the artists picked up stones presenting some resemblance to the outlines of the human head and face, and that they heightened this resemblance by labour. No flint weapons or tools have been found in the same pits with the stones; and the question for experts to decide is, presumably, whether any of them bear distinct traces of human labour.

### Religions and Race Suicide.

Political economists have been much exercised in their minds of late years over the decadent birth-rates in those nations who may be considered as the most advanced in intelligence and civilisation, and a great deal has been said about over education and race suicide. Among the more recent theories put forward regarding the factors which tend to produce the phenomenon is one which involves the different creeds. The respective reproductiveness of the various religious communities was worked out for a certain district in New York and the result is very interesting in spite of the fact that no definite conclusions can be based upon the figures as they stand. It was found for example that the average number of children in the Protestant families was 1·85, in the Roman Catholic 2·03, and in the Hebrew 2·54. Beside this it was noticed that only 16 6 per cent. of the Hebrews were without children while the figure for Protestants was 28 3 per cent. Moreover, six times as many Hebrew families have nine children as have the Protestant families. Among the different Protestant sects there are some which show a higher average of reproductiveness than the Roman Catholics; for example, the Episcopalian and Presbyterian. It is noteworthy that the Protestants who specified the particular sect to which they adhered showed a higher average of children than those who pronounced themselves to be Protestants but of no particular sect. These latter, again, exhibited greater apparent fertility than those who called themselves agnostics. These facts supply ample material for meditation, and it would be possible to philosophise upon them to any extent, but it should be remembered that the figures quoted require a good deal of adjustment before they can be absolutely relied upon. For instance, they are based upon the number of living children in each family, and a correction would have to be made concerning the number of children who have died. It is quite conceivable that the infantile mortality may differ considerably among the different religious communities, but, although this may be the case to some extent, it is hardly likely to account entirely for the differences. There is, again, the question of racial fecundity to be considered, and the figures mentioned might need to be further modified accordingly. It would be of the greatest interest to know what is the true relation, if there be any, between religion and a falling birth-rate, so that efforts might be properly directed to counteract the present-day tendency towards race suicide.

### Hæmaturia due to Bilharzia Hæmatobia.

Recent events in South Africa are probably responsible for the increased number of cases of this condition which have been observed during the last year or two in this country. Numerous instances have lately been reported both from private and hospital practice. The parasite is widely distributed over the African continent, and is particularly common in Egypt, on the West Coast, and in certain parts of Cape Colony, Natal, and the Transvaal; and in these countries it is responsible for the widely-prevalent endemic hæmaturia. There is reason to believe that infection occurs during bathing, the embryos either penetrating the skin or entering through the anus or urethra. The mature worms are found principally in the small veins of the portal system and of the bladder and kidneys. Numbers of the ova penetrate the vessels and escape through the mucous membranes, more particularly those of the bladder and rectum. Hence one of the commonest evidences of their presence is the escape of blood in the urine. Indeed, it is a common experience that, with the exception of more or less pronounced anæmia and consequent weakness, hæmaturia is the sole ground of complaint. In some instances there is in addition more or less pelvic pain. The blood is usually limited to the last stage of micturition, but it may discolour the entire volume of the urine. The diagnosis is readily made if the urinary deposit is placed under the microscope. The ova are then readily recognised. They are bright, translucent, oval bodies, having a well-defined outline. Each at one extremity shows a short, sharply-pointed spike or spine, though occasionally the spine is situated laterally. Even in the slightest form the condition is difficult to treat with success. The parent worm, from its position in the blood-vessels, is beyond the reach of the ordinary anthelmintics, though at the recent Medical Congress at Cairo it was stated that the hæmaturia could be controlled, at least to some extent, by extract of male fern. Another risk is nephritis, and the detection of tube-casts or other renal elements in the deposit will seriously aggravate the prognosis.

